# Idiopathic pulmonary vein thrombosis treated with apixaban

**DOI:** 10.1002/rcr2.803

**Published:** 2021-07-21

**Authors:** Dallis Ngo, Ghulam Aftab, Arjun Madhavan, Amar Bukhari

**Affiliations:** ^1^ Division of Pulmonary, Critical Care and Sleep Medicine Saint Peter's University Hospital New Brunswick NJ USA

**Keywords:** Apixaban, breast cancer, idiopathic, pulmonary vein thrombosis, venous thrombosis

## Abstract

Pulmonary vein thrombosis (PVT) is a rare clinical finding that is potentially fatal and with an unknown incidence rate as known cases exist predominantly in case reports. We present the case of a 58‐year‐old female who reported sudden onset of chest pain, shortness of breath, and dyspnoea on exertion. A computed tomography (CT) pulmonary angiogram was negative for evidence of pulmonary embolism; however, it did demonstrate the evidence of thrombosis of the right lower lobe segmental pulmonary vein. She had no identifiable aetiologies for her PVT; therefore, she was diagnosed with idiopathic PVT and was treated successfully with apixaban. This case represents the 14th incidence of idiopathic PVT in the current body of medical literature and the first case of successful treatment with apixaban.

## Graphical abstract

Idiopathic pulmonary vein thrombosis (PVT) is exceedingly rare with an unknown incidence rate as it has only been reported in case reports. We present a case of idiopathic PVT which represents the 14th identified case within the current body of medical literature and the first ever case successfully treated with apixaban.

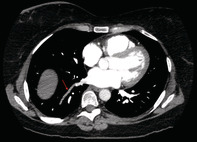

## Introduction

Pulmonary vein thrombosis (PVT) is a rare and life‐threatening condition commonly associated with interventional thoracic procedures such as thoracic surgery and radiofrequency catheter ablation for atrial fibrillation. Idiopathic PVT is exceedingly rare with an unknown incidence rate as it has only been reported in case reports. We present a case of idiopathic PVT which represents the 14th identified case within the current body of medical literature and the first ever case successfully treated with apixaban.

## Case Report

We present a 53‐year‐old female with a past medical history significant for right breast cancer, status‐post lumpectomy followed by completion of six rounds of chemotherapy (Taxotere [Sanofi‐Aventis, France], Herceptin [Genentech, USA], and carboplatin) and 36 rounds of radiation six years prior and currently in remission, hypertension, and bilateral lower extremity varicose veins, status‐post phlebectomy 14 years prior complicated by a post‐operative right lower extremity deep vein thrombosis treated successfully with three months of Coumadin (Bristol Myers Squibb, USA). She presented to the hospital with complaints of sudden onset sharp, constant, and non‐radiating substernal chest pain while eating dinner with her husband at home. Her symptoms were not associated with nausea, diaphoresis, haemoptysis, palpitations, or syncope. She denied any symptoms suggestive of acid reflux; however, she did endorse shortness of breath and dyspnoea on exertion. There were no provoking factors. She denied any recent history of recent immobilization, trauma, calf/leg pain or swelling, long car rides, or air travel. There was no surgical history of prior lobectomies, lung transplants, or atrial fibrillation ablations. Her family history is negative for blood dyscrasias or blood clots and her social history is negative for tobacco or illicit drug use. She has no active cancer disease.

**Figure 1:**
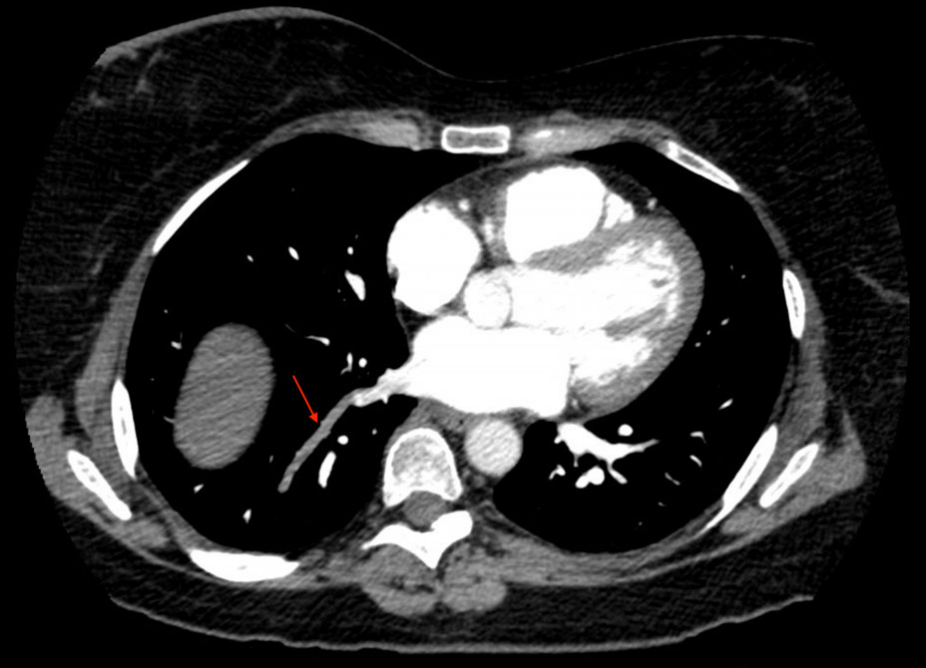
Axial view of a computed tomography (CT) pulmonary angiogram demonstrating a pulmonary vein thrombus (red arrow) involving the right lower lobe segmental vein.

Her initial investigations in the emergency department demonstrated completely unremarkable parameters in regards to her complete blood count, complete metabolic panel, prothrombin time, troponin‐I, d‐dimer, and electrocardiogram. Her physical examination was unrevealing. The chest radiograph did not demonstrate evidence of acute disease. A computed tomography (CT) pulmonary angiogram was performed which did not demonstrate evidence of pulmonary arterial embolism; however, there was evidence of significant thrombosis of a right lower lobe segmental pulmonary vein without an obvious visualizable aetiology (Fig. 1). Her transthoracic echocardiogram demonstrated normal left ventricular systolic function without evidence of right heart strain or elevated right ventricular systolic pressure. There was no evidence of mitral stenosis and no presence intra‐atrial or ventricular thrombus formation. A hypercoagulable workup, consisting of anti‐cardiolipin IgG/M, lupus anticoagulant, and proteins C and S, was performed and found to be negative. No identifiable source was determined to be the underlying culprit; therefore, her pulmonary vein thrombus was determined to be idiopathic in nature.

The rest of her hospital course was unremarkable after the initiation of intravenous therapeutic anticoagulation via unfractionated heparin. She was discharged home with instructions to continue apixaban for three months followed by re‐evaluation in the outpatient setting. During her follow‐up visit in our outpatient pulmonary clinic, she was found to be in good health with complete resolution of all prior presenting symptoms and normoxic on ambient air. A follow‐up CT scan was offered to evaluate for resolution of the thrombus; however, due to the risk of further radiation, the patient declined.

## Discussion

PVT is a rare clinical condition with an unknown incidence rate as majority of our current understanding stems from case reports. Due to the non‐specific nature of its presentation, it presents a diagnostic challenge. Patients may demonstrate evidence of dyspnoea, pleuritic chest pain, cough, or haemoptysis which may lead to the misdiagnosis of a pulmonary embolism. Clinically, there is no difference in patient presentation of PVT in a provoked scenario and idiopathic [1]. The rare occurrence of identified cases in the current literature could be explained by the extensive collateral venous network within the pulmonary circulation, although, if left untreated, it could potentially result in interstitial pulmonary oedema, right ventricular failure, and pulmonary infarction [1, 2]. Dyspnoea from PVT is not always conducive to an abnormal resting echocardiogram as demonstrated by prior reported cases in Table 1. Such a large pulmonary vein thrombus can cause a reduction in total pulmonary vascular volume with associated decrements of left atrial volume and cardiac output to meet exercise demands [15, 16]. This may be more evident with a more invasive exercise right heart catheterization.

**Table 1:** Idiopathic PVT cases reported in the current literature.

Author	Age and gender	Comorbidities	Clinical presentation	Method of diagnosis and result	Additional workup	Treatment
Selvidge and Gavant, 1999 [3]	33 F	Sickle cell trait, tobacco smoker, cocaine	Acute onset left‐sided abdominal pain, nausea, and vomiting	CT abdomen with contrast: irregular, small areas of non‐enhancing infarction within the spleen and a 2‐cm diameter filling the defect in the left atrium extending from a thrombus in the distal right lower pulmonary vein	CXR: patchy opacity in the right lower lobe TTE: normal findings ECG‐gated MRI: bland thrombosis of the right lower pulmonary vein with extension into the left atrium	Oral anticoagulant
Alexander et al., 2009 [2]	47 F	None	Massive haemoptysis, left chest pain, dyspnoea	Pathology of the left lower lobectomy: sections of the lung parenchyma demonstrated red hepatization with thrombosis of the pulmonary venous system	TTE: no evidence of thrombosis in the left atrium	Surgical resection of the affected lobe and thrombectomy
Komatsu et al., 2011 [4]	57 M	Hyperlipidaemia	Chest pain with myocardial infarction	CT chest: bilateral lower pulmonary vein thrombus		Warfarin. Antiplatelet for CAD
Mumoli and Cei, 2012 [5]	80 M	Coronary artery disease s/p CABG, congestive heart failure	Acute shortness of breath	CT chest: bilateral pleural effusions and a large thrombus in the left superior pulmonary vein	CXR: near‐round opacity in the upper left lobe with fissure involvement TTE: ejection fraction 30% Hypercoagulable workup: normal except homocysteine of 18.5 μmol/L	Enoxaparin bridged to warfarin
Takeuchi, 2012 [6]	79 M	Hypertension	Chest pain	64‐MDCT: 17.2 × 1.2 × 1.3 mm thrombus was situated at the proximal side of the left upper pulmonary vein and calcification of the left anterior descending artery		Warfarin
Wu et al., 2012 [7]	30 M	Hypertension	Chest pain	CT chest PE protocol: multifocal consolidation and ground‐glass opacities in the left lower lobe, left‐sided effusion, well‐defined filling defect, and occlusion within a left inferior pulmonary vein and homogenous hypodense attenuation in the left atrium after contrast administration	d‐dimer: normal Thrombophilia workup (antithrombin III, protein C/S): negative Tumour markers (CEA, AFP, CA19‐9, CA‐125, NSE): negative TEE: 2 cm diameter filling defects in the left atrium suggestive of thrombus	Unknown
Takeuchi, 2013 [8]	73 M	Hyperlipidaemia, asthma	Chest pain	64‐MDCT: no coronary artery stenosis. Thrombus in the left upper pulmonary vein	CXR: normal d‐dimer: <0.5 μg/mL Protein S activity: 96% Protein C activity: 131%	Dabigatran 150 mg q12 h
Takeuchi, 2013 [9]	70 M	Coronary artery disease	Chest pain	64‐MDCT: large thrombi in the left lower pulmonary vein expanding into the left atrium	CXR: normal TTE: thrombus in the left atrium 30.2 mm × 8.1 mm, no thrombus in left atrial appendage	Aspirin 100 mg
Takeuchi, 2014 [10]	68, M	Hypertension, hyperlipidaemia, stroke	Chest pain	64‐MDCT: calcification of the coronary arteries. A thrombus in the right lower pulmonary vein	CXR: normal d‐dimer: 0.5 μg/mL Protein S activity: 66% Protein C activity: 155%	Dabigatran
Takeuchi, 2015 [11]	82 M	Hypertension, hyperlipidaemia	Chest pain	64‐MDCT: thrombus in the right lower pulmonary vein	d‐dimer: 0.5 μg/mL Protein S activity: 85% Protein C activity: 107%	Dabigatran
Rana et al., 2016 [12]	63 M	None	Chest pain	CTPA: no pulmonary embolism. Thrombus in the pulmonary vein extending into the left atrium	CXR: normal d‐dimer: 1800 ng/mL TTE: normal TEE: confirmed PVT. Pulmonary artery systolic pressure 28 mmHg Thrombophilia workup: normal Tumour markers (AFP, beta‐2 microglobulin, CA 19‐9, PSA): normal	Low‐molecular weight heparin bridged to warfarin
Patel et al., 2017 [13]	77 F	Carcinoid tumour s/p resection, hypertension, hyperlipidaemia	Acute shortness of breath	CTA chest: no pulmonary embolism. Non‐exclusive thrombus in the inferior	TTE: positive McConnell's sign Thrombophilia workup (factor V Leiden, protein C/S): normal Lower extremity duplex US: normal	Rivaroxaban
Barreiro et al., 2018 [14]	26 F	Gravida 5 para 5	Chest pain	CTA chest: right hilar mass or lymph node causing encasement of the right main pulmonary artery and infiltrates in the right middle and lower lobes, consistent with PVT	CXR: bilateral infiltrations in lower lobes in an interstitial pattern ANA, lupus anticoagulant, and C‐ANCA: negative P‐ANCA: elevated	Oral anticoagulant
Ngo, 2021 (current case)	53 F	Right breast cancer in remission, varicose veins	Chest pain, shortness of breath	CTPA: significant thrombosis of the right lower lobe segmental pulmonary vein	CXR: normal d‐dimer: normal Anti‐cardiolipin, lupus anticoagulant, protein C/S: normal	Apixaban

AFP, alpha‐fetoprotein; ANA, antinuclear antibody; C‐ANCA, antineutrophil cytoplasmic antibodies; CABG, coronary artery bypass graft; CAD, coronary artery disease; CEA, carcinoembryonic antigen; CT, computed tomography; CTA, computed tomography angiography; CTPA, computed tomography pulmonary angiogram; CXR, chest X‐ray; ECG, electrocardiogram; MDCT, multidetector computed tomography; MRI, magnetic resonance imaging; NSE, neuron‐specific enolase; P‐ANCA, perinuclear anti‐neutrophil cytoplasmic antibodies; PE, pulmonary embolism; PSA, prostate‐specific antigen; PVT, pulmonary vein thrombosis; s/p, status post; TTE, transthoracic echo; US, ultrasound.

Identified causes of PVT are usually associated with manipulation of the pulmonary veins during lung transplantation or lobectomy, radiofrequency ablation for atrial fibrillation, sclerosing mediastinitis, pulmonary malignancy, atrial myxoma, congenital pulmonary vein narrowing, or mitral stenosis [17]. In a review of five cases of idiopathic PVT, four cases were found to have normal d‐dimer levels in addition to 17 of 23 cases of PVT with an identifiable cause, suggesting that d‐dimer does not appear to be correlated with the presence of a PVT [13].

There is no gold standard for the diagnosis of PVT. Diagnosis is often made with the use of a combination of multiple imaging modalities such as transoesophageal echocardiogram, cardiac gated magnetic resonance imaging, pulmonary angiogram, or CT pulmonary angiogram [14]. CT scans that are typically meant to evaluate the pulmonary arterial anatomy may be misleading due to the poor opacification and mixing artefact in the pulmonary vein, potentially mimicking thrombus and left atrial masses. Utilizing longer scan delays allows for better evaluation of both pulmonary veins and left atrium [1]. Although our patient did not receive a repeat CT scan to demonstrate resolution, citing risk of radiation, it is not standard of practice to do so if the patient's symptoms have resolved as clot resolution occurs in as little as three weeks with anticoagulant treatment [18].

To date, there have been no randomized control trials evaluating treatments for PVT; however, multiple case reports have demonstrated success with the use of warfarin, rivaroxaban, and dabigatran [13, 14]. Our case is rare in that it represents the 14th case (Table 1) of a reported idiopathic PVT and the first reported treated successfully with apixaban.

### Disclosure Statement

Appropriate written informed consent was obtained for publication of this case report and accompanying images.

### Author Contribution Statement

All authors listed were directly and equally involved in the conceptualization, literature review, and writing of this manuscript.
